# Effect of sound insulation on noise reduction in an agricultural tractor cab

**DOI:** 10.1038/s41598-022-26408-3

**Published:** 2022-12-21

**Authors:** Hyun-Woo Han, Won-Hong Im, Hyo-Joon Choi, Seung-Je Cho, Sang-Dae Lee, Young-Jun Park

**Affiliations:** 1 Department of Biosystems Engineering, Seoul National University, Seoul, 08826 Republic of Korea; 2 Convergence Major in Global Smart Farm, Seoul National University, Seoul, 08826 Republic of Korea; 3 Samwoo Agri-machinery Co., Ltd., Daegu, 42710 Republic of Korea; 4 Korea Institute of Industrial Technology, Gimje, 54325 Republic of Korea; 5 Department of Bioindustrial Machinery Engineering, Jeonbuk National University, Jeonju, 54896 Republic of Korea; 6 Research Institute of Agriculture and Life Sciences, College of Agriculture and Life Sciences, Seoul National University, Seoul, 08826 Republic of Korea

**Keywords:** Mechanical engineering, Acoustics

## Abstract

Tractor cab interior noise is a risk factor that degrades operators’ work performance and threatens their health; therefore, the noise must be reduced to ensure farmworkers’ safety and efficiency. Cab interior noise can be classified as structure-borne noise and air-borne noise. Structure-borne noise has been extensively studied. However, although air-borne noise greatly contributes to cab interior noise, detailed frequency-domain analyses have not been performed. In this study, the components of cab interior noise were identified in the frequency domain through an order analysis, which helped improve the sound insulation of the cab and reduce the effects of air-borne noise. A test was performed while driving a tractor on a chassis dynamometer in a semi-anechoic chamber for reproducible measurement and evaluation. The A-weighted sound pressure was transformed by a fast Fourier transform algorithm, and its order was tracked by the engine speed signal. In addition, a direct path was identified by acoustic images using a sound camera. The contributions of major noise sources were identified through an order analysis. We proved that air-borne noise significantly contributes to the interior noise of tractor cabs and that improvement of the cab sound insulation is an effective noise-reduction technique.

## Introduction

The high noise level of agricultural machinery during operation is a significant risk factor that reduces the performance of farmworkers and threatens their health. The Occupational Safety and Health Administration (OSHA) has reported that prolonged exposure to high noise levels can cause physical disability and mental illness in workers^1^. According to Lashgari and Maleki, farmworkers have higher rates of hearing loss than workers in most other occupations^2^. It has also been reported that exposure time and changes in tractor cab interior noise have a significant impact on the operator’s performance^3^. Therefore, research on noise reduction of agricultural machinery must be conducted to improve farmworkers’ work environment and efficiency and to prevent accidents.

The cab in tractors and combines serves the purpose of preventing rollover accidents and constructing a comfortable working environment. In particular, the noise level at the driver’s ear position is lower in a tractor with a cab than that in a cabinless (open-station) tractor^4^; this is because the cab reduces the radiation noise of the engine, intake and exhaust system, and transmission system by blocking the direct paths between the cab and the exterior. In addition, the cab installation rate has continuously increased owing to the advantages of reducing the impact of weather conditions and providing thermal comfort^5^.

The interior noise level of a cab is an important performance indicator of agricultural tractors. The interior noise has four components: structure-borne noise, air-borne noise, internal-sources-radiated noise, and reverberation noise^6^. Structure-borne noise refers to the noise radiated from the cab structure owing to vibrations generated from the engine and transmission system. It mainly dominates in the frequency range below 250 Hz and causes severe noise events, such as booming^7^. The noise radiated from external sources and transmitted into the cab is called air-borne noise. Exhaust noise is an example of an air-borne noise in a tractor. It is reduced by the Helmholtz resonator in the muffler. Since the high-speed grazing flow can degrade the damping performance of the Helmholtz resonator^8^, the design of the muffler is also important from the perspective of the acoustic source^9^. The air-borne noise is dominant in the high-frequency range; hence, air-borne noise contributing to the interior noise can be effectively mitigated through the improvement in cab sound insulation and the utilization of acoustic materials^10^ from the perspective of transfer path. Introducing changes in the noise transfer path is a key tool when the characteristics of the source cannot be improved^11^. The third type is the noise generated by internal sources. Finally, the reverberation noise is caused by reflection of the above three types of noise off the hard surfaces in the cab.

Structure-borne noise can cause severe noise problems such as booming during operation according to the dynamic characteristics of the cab. Most studies have mainly focused on the coupled system between the cab structure and the acoustic cavity. Mohammad et al. investigated boom noise by optimizing the stiffness of the cab bottom panel using a finite-element model^12^. Velioglu et al. developed a vibro-acoustic analysis model of a tractor cab and proposed a correlation method to improve the accuracy of the model, using transfer path analysis and experimental modal analysis^13^. Li and Zhao evaluated the panel contribution of tractor cab interior noise using a finite-element model^14^. Zhang et al. conducted a panel contribution analysis on a heavy commercial vehicle cab and optimized the panel thickness using a genetic algorithm^15^. As mentioned above, experimental, numerical, and hybrid experimental–numerical methods have been studied in order to mitigate the most severe noise such as resonance or boom noise in the low-frequency band, where structure-borne noise is often dominant during operation.

Air-borne noise is recognized as the dominant component of cab interior noise in the high-frequency range. The noise in the high-frequency range is usually transferred through the air-borne path, holes, and through-paths; therefore, sound insulation using proper acoustic barriers is crucial^16^. Velioglu et al. reported that air-borne noise significantly contributes to the cab interior noise, and the sound insulation of the cab is highly essential for noise reduction^13^. In addition, Ryland and Turnquist reported that a poor-quality cab may further increase the noise level, and that the noise is effectively reduced only when proper sound insulation using acoustic materials is applied^17^. Wang et al. showed that the a coupled resonant acoustic material installed between the cab and engine room resulted in effective noise reduction in the mid- and high-frequency range^18^. However, existing studies mainly dealt with the improvement of the overall level and could not identify the components constituting the cab interior noise (e.g., dominant frequencies or order components). In addition, the effect of improved sound insulation and sound-absorbing material attachment on the air-borne noise in the cab could not be closely analyzed in the frequency domain. It has been frequently underlined in the literature that the cab sound-insulation performance and air-borne noise insulation are essential for noise reduction. Nonetheless, causes and effects have not been identified and appropriate acoustic treatments have not been established.

The purpose of this study is to evaluate the interior noise of the tractor cab and analyze the frequency characteristics of dominant noise components, as well as to identify the noise-reduction effect of the improvement to cab sound insulation, especially for air-borne noise. The paper is organized as follows: Method section details the tractor driving test in the semi-anechoic chamber and sound pressure measurements at the driver’s ears, and the results of the frequency and order analysis of the cab interior noise and the direct-path insulation are presented in Result and Discussion section. Finally, conclusions are presented.

## Methods

### Tractor driving test in the semi-anechoic chamber

**Figure 1:**
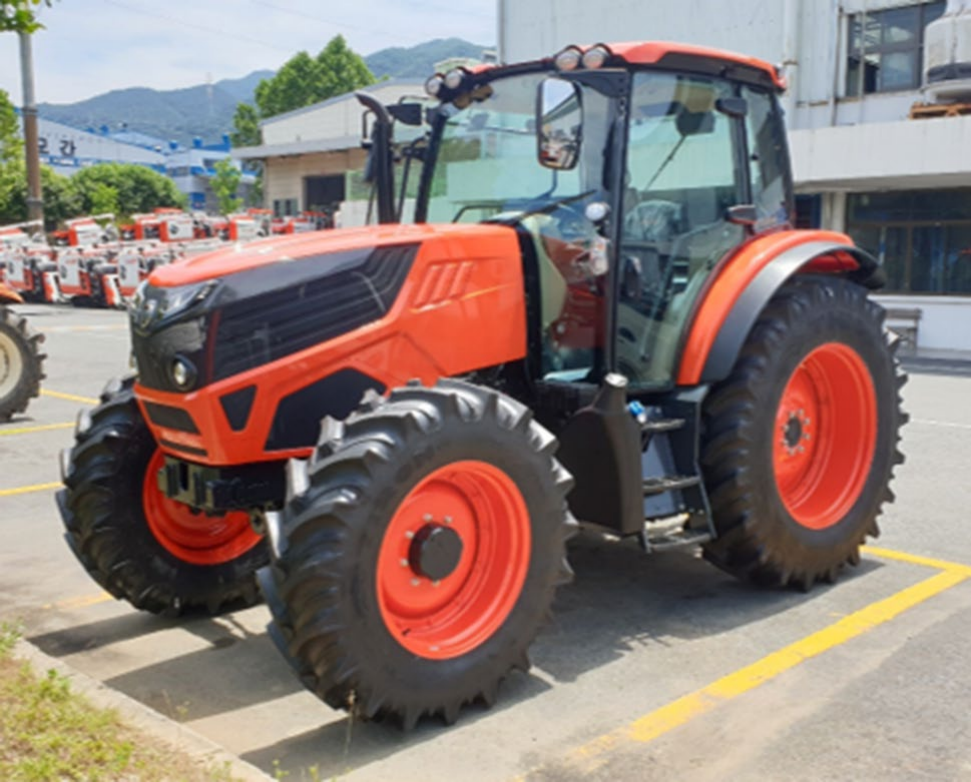
Prototype tractor used in this study.

A 104.5 kW class tractor, shown in Fig. 1, was used in this study. The transmission system consists of an eight-speed main shift part capable of power-shifting by wet multi-plate clutches, a two-speed sub-shift part of the constant-mesh type, and a creep mode. Power is transmitted by a transfer case to the front axle capable of four-wheel drive, which enables narrow radius turns since it is composed of two speeds. Detailed specifications of the tractor are listed in Table 1.

**Table 1:** Tractor specifications.

**Dimension**	
Length (mm)	4620
Width (mm)	2527
Height (mm)	2755
Weight (kg)	4356
Wheel base (mm)	4600
Minimum ground clearance (mm)	470
**Transmission**	
Main part	
Shift type	Wet multi-plate clutches
Stages	8 stages
Sub-part	
Shift type	Constant mesh
Stages	2 stages
**Steering**	
Type	Hydraulic
**Tire**	
Front	380/85R28
Rear	460/85R38
**Engine**	
Type	4-cylinder diesel
Power (kW)	104.5
Exhaust volume (L)	3.833
Turbocharger type	Exhaust turbocharger

**Figure 2:**
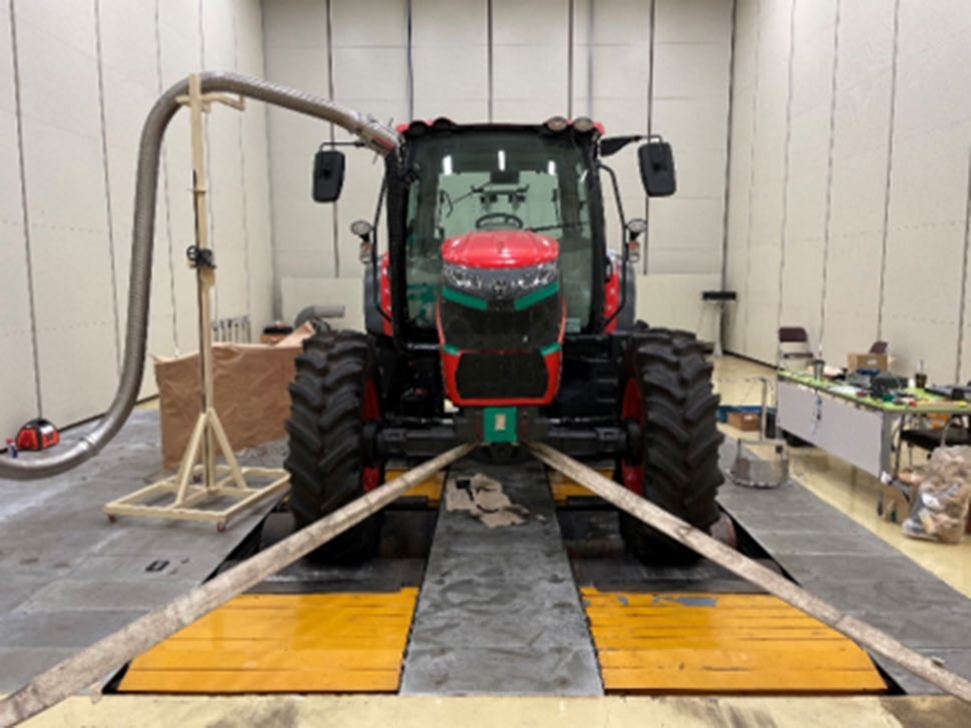
Test tractor on the chassis dynamometer in the anechoic chamber.

The cab interior noise was evaluated during engine run-up conditions driving on the chassis dynamometer in all 16 forward gears. Only the tractor’s acceleration load was applied without any external load to the chassis dynamometer. Reverse and creep modes, which have a low frequency of use and low rotational speed, were excluded from the evaluation. The test was performed on a chassis dynamometer in a semi-anechoic chamber, as shown in Fig. 2, to ensure reproducibility and repeatability and to block the influence of external noise. The background noise level in the semi-anechoic chamber was approximately 25 dB(A), and the temperature was kept constant at approximately 20 °C by an air conditioner. Detailed specifications of the chamber are listed in Table 2.

**Table 2:** Semi-anechoic chamber specifications.

Item	Specification	
Dimension (width × length × height)	18 m × 9 m × 7 m	
Background noise	Stationary	20 dB(A)
	HVAC operation	25 dB(A)
Cut-off frequency	63 Hz (according to ISO 3745)	
Natural frequency	8 Hz	
Absorption material	Broad compact absorber (BCA)	

The order of the selected noise sources referenced to the engine speed was calculated, as shown in Table 3. The order of each component was calculated using specifications such as the number of gear teeth, number of fan blades, and number of tire lugs.

**Table 3:** Noise source and order information of the test tractor.

Source	Component	Order
Engine	Main order	2
	Fan	11.6
Transmission	Main shift	40
	Transfer case	48.7
Axle	Front differential	13.91
	Front final reduction	4.35
	Rear differential	13.91
	Rear final reduction	3.62
Hydraulic pump	Input gear	43
Tire	Front No. of lugs: 19	1.38
	Rear No. of lugs: 21	1.16

### Measurement of sound pressure level at the driver’s ears

**Figure 3:**
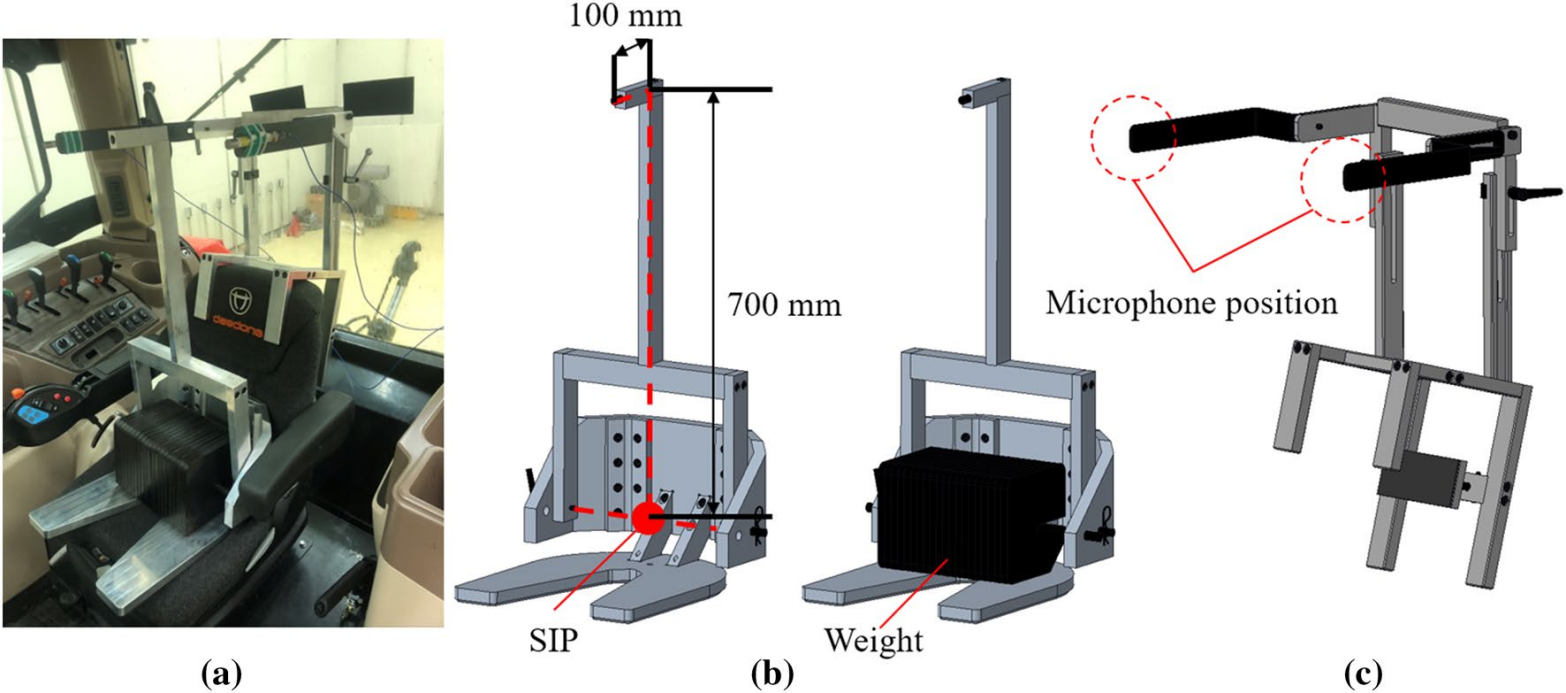
Noise measurement setup: (**a**) test jigs on the operator’s seat, (**b**) test jig for determining SIP, and (**c**) test jig for determining microphone position.

To enable reproducible noise measurement at the driver’s ear position, the seat index point (SIP) was determined and a testing device designed to position the microphone at the driver’s ear position was used according to ISO 5353 and ISO 5131, respectively^19,20^. The microphones were installed 700 mm above, 100 mm in front, and 250 mm in the lateral direction of the SIP, as shown in Fig. 3^19^.

As shown in Fig. 3b, the SIP was determined using the testing device proposed in ISO 5353^19^. The total weight of the device was 65 kg, and the seat suspension was fixed in a neutral position. The microphones were fixed with the diaphragm facing forward using the device shown in Fig. 3c, which is installed on the driver’s seat backrest.

**Figure 4:**
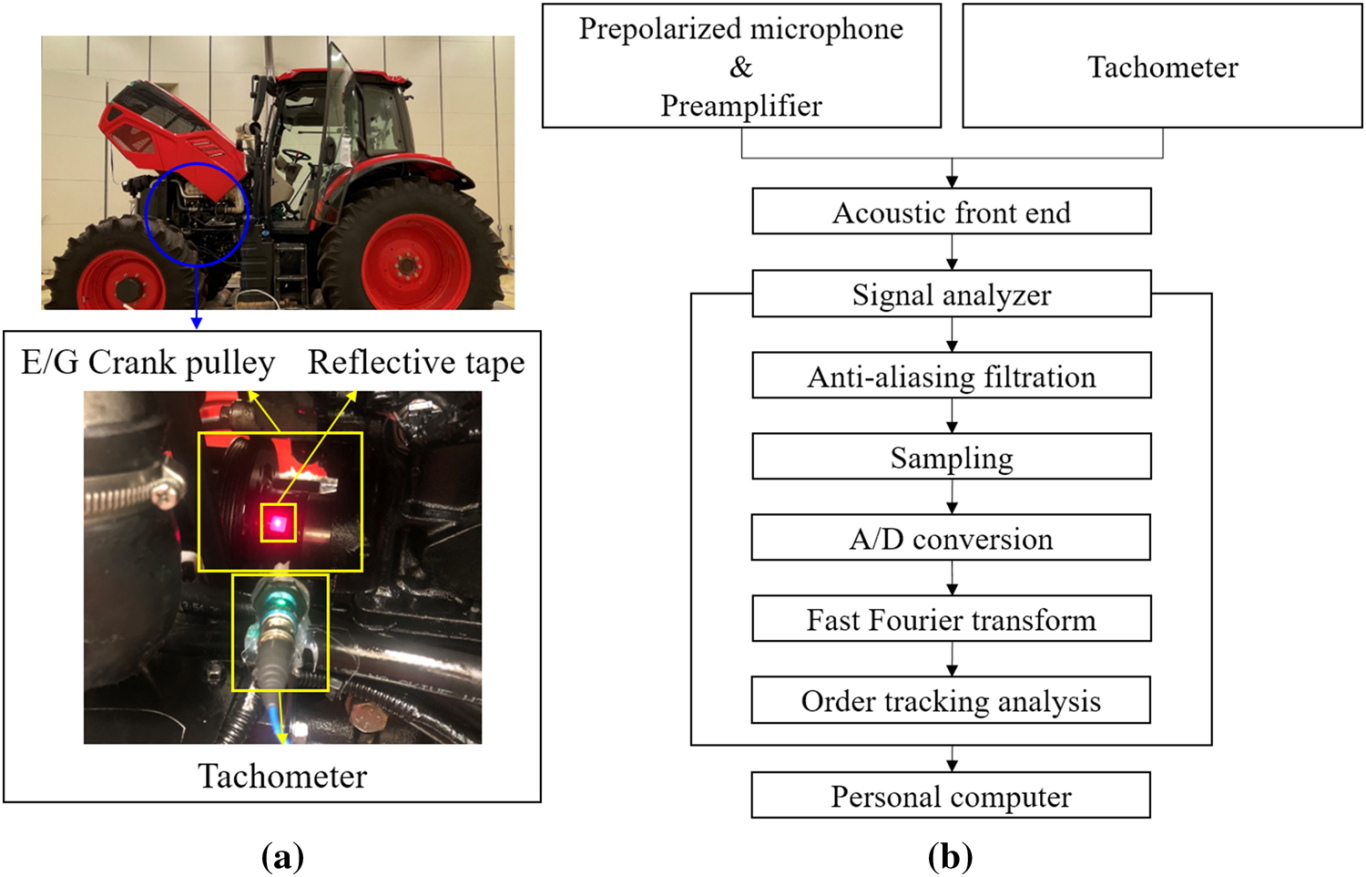
Installed sensor and signal processing flow chart: (**a**) tachometer installed at the engine crank pulley to measure engine speed, and (**b**) flow chart of sound signal processing.

A tachometer was installed at the crank pulley to enable order tracking and color map analysis according to the engine speed as shown in Fig. 4a. The acoustic signal was measured and analyzed, as shown in the flow chart in Fig. 4b. Gathered signals were analyzed using Simcenter Testlab 2019 commercial software (Siemens, Leuven, Belgium). Since the main purpose of this paper is to reduce the noise perceived by humans, A-weighting was applied to the sound pressure level of all results. The A-weighted sound pressure was transformed by a fast Fourier transform (FFT) algorithm, and its order was tracked by the engine speed signal. The specifications of the sensors, data acquisition system, and sound camera used in the test are listed in Table 4.

**Table 4:** Specifications of equipment used for cab interior noise measurement and analysis.

Equipment	Model	Specification
Frontend	Siemens SCADAS Mobile	Maximum sampling rate: 204.8 kHz Dynamic range: 150 dB ADC technology: 24-bit delta-sigma
Microphone	PCB 377B02	Nominal diameter: 1/2″ (12.7 mm) Frequency range: ($\pm$ 2 dB) 3.15 to 20,000 Hz Dynamic range: 147 dB(A)
Preamplifier	PCB 426E01	Temperature range: − 40 to 80 °C Output voltage: $\pm$ 7 Vpk
Acoustic calibrator	Larson Davis CAL200	Operating frequency: 1000 Hz Sound pressure level: 94 or 114 dB(A) Distortion: less than 2%
Tachometer	PCB LT-2	Maximum speed: 100,000 rpm Operating range: 51 cm
Sound camera	SM instruments SeeSV-S206	96 Ch. Digital MEMS microphone Frequency range: 25–12,800 Hz Signal to noise ratio: 64 dB(A)

## Results and discussion

The first section presents the interior noise of the tractor cab according to the gear stages, and the results of order analysis based on the system information shown in Table 3. The sound pressure level in narrow frequency band according to the orders in Table 3 was tracked by engine speed signal. The contributions of each sound source were calculated as the sum of the sound pressures of relevant order. In addition, in order to examine the effective use of acoustic materials in the high-frequency range, the noise contributions of the high- and low-frequency bands based on 500 Hz were also compared.

In the second section, the direct path between the cab and the exterior was determined by visual identification and the sound camera. The identified direct paths were insulated with grommets and steel plates. Additionally, sound absorption material was applied to the front panel of the cab facing the engine room. The paths identified by sound camera were insulated using sealing material. Blocking the direct paths between the cab and the exterior was confirmed to have noise-reduction effects.

### Frequency and order analysis of cab interior noise

**Figure 5:**
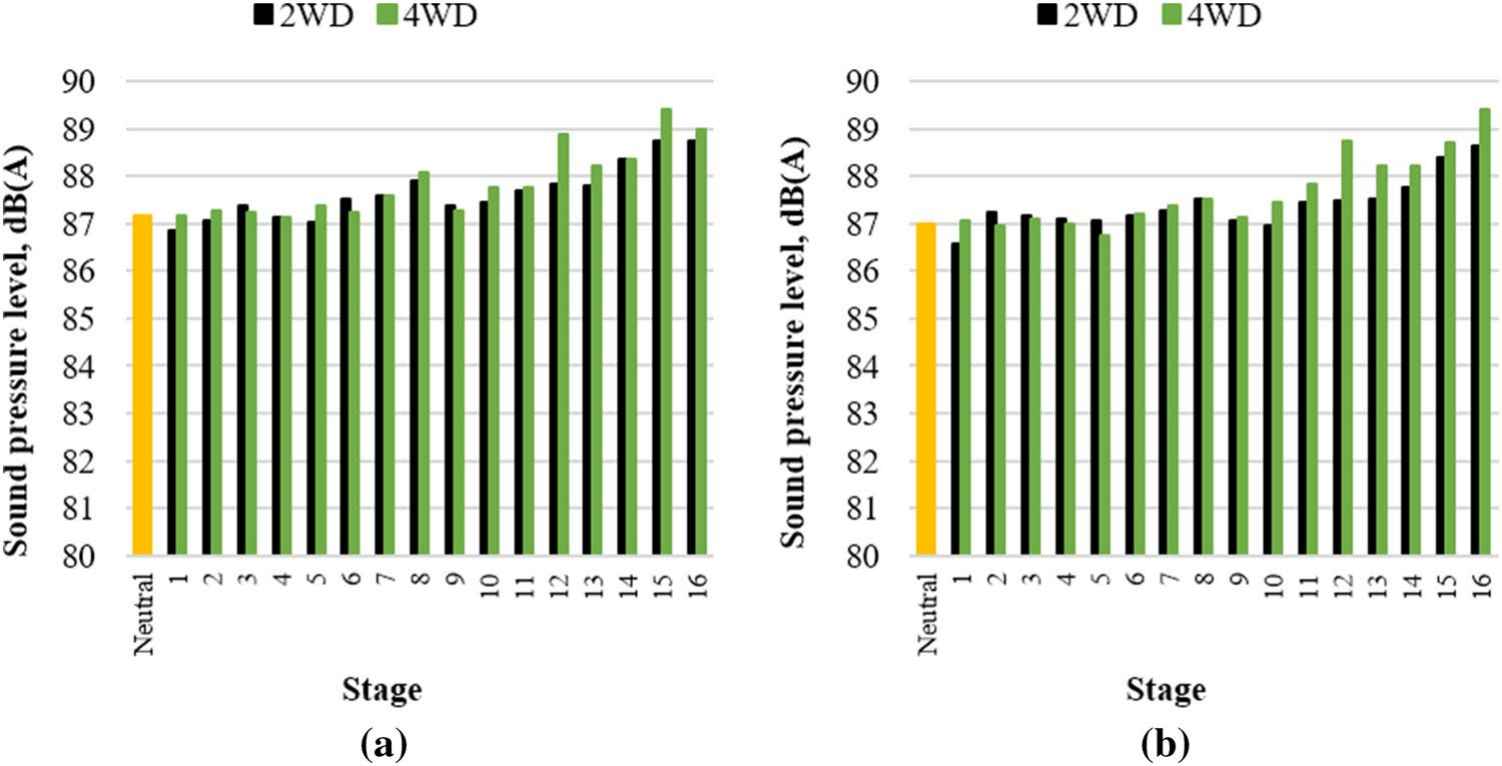
Maximum overall noise level of the initial state: (**a**) operator’s left-ear position and (**b**) operator’s right-ear position.

The maximum sound pressure level for each stage was 87–89 dB(A), as shown in Fig. 5. The higher the gear stage the higher the overall noise level, which is known to be because both the powertrain and tire noise increase as the vehicle speed increases^21^. In all stages, the noise level was lower than the OSHA’s 8-h exposure limit of 90 dB(A)^1^. However, considering the noise increase due to the operational load, it is not completely safe from the risk of hearing loss due to long-term noise exposure. In addition, a noise level lower than 75 dB(A) is required for occasional telephone or radio use according to military standards^22^. Therefore, the noise level in the tractor cab used in this study should be reduced.

The main sources of tractor cab interior noise were engine harmonic orders, intake and exhaust noise, gear whine noise of transmission and input gear of hydraulic pump, and tire lug noise. Because the noise components (except the transmission noise) are independent of the gear stage, the case of the operator’s right ear at stage 16, which generated the largest noise level, was analyzed in detail. In the case of other stages, only the gear mesh frequency (GMF) changed according to the selected gear, and the noises from the engine, pump, and tires remained the same.

**Figure 6:**
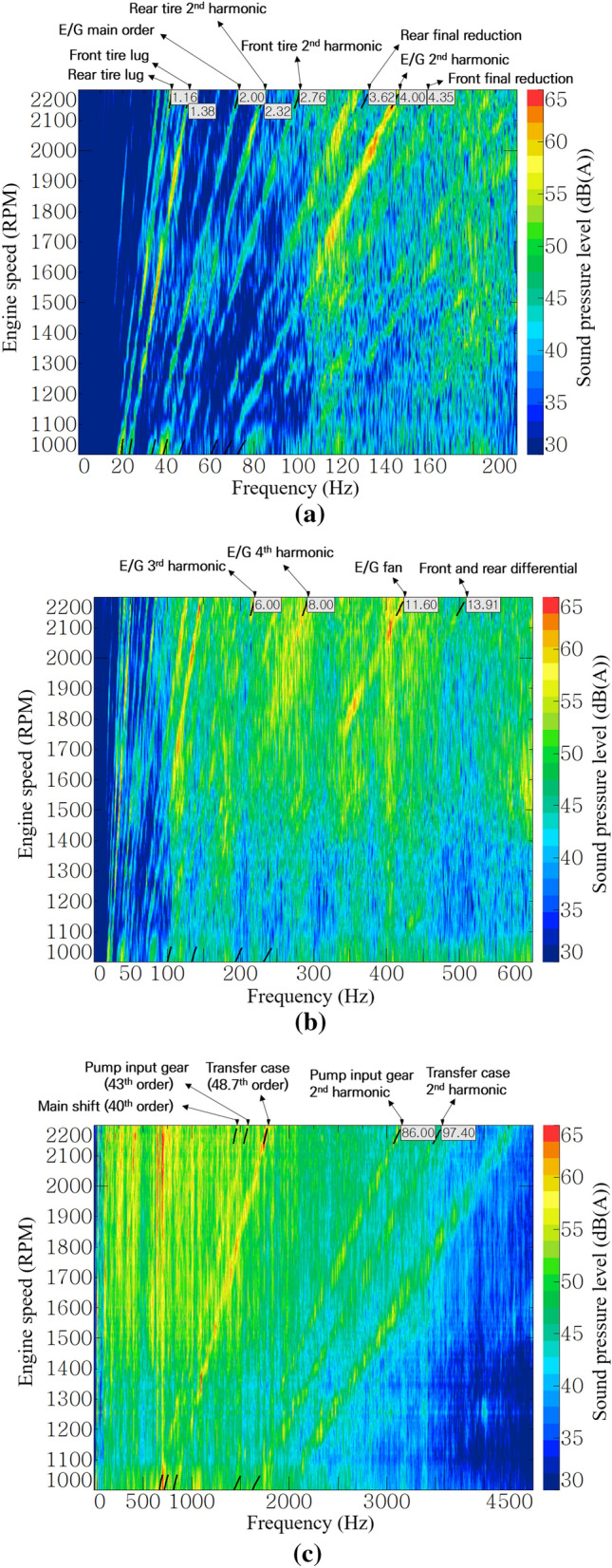
Noise color map of stage 16 at operator’s right-ear position: (**a**) up to 200 Hz, (**b**) up to 600 Hz, and (**c**) up to 4500 Hz.

Figure 6 show the noise color maps of the operator’s right ear at stage 16. Each color map had a different frequency range for better resolution. The low-order noise below the 200 Hz range in Fig. 6a was dominated by the influence of engine explosion frequency and its harmonic orders and tire lugs. The noise order of the final reduction gear (planetary gear set) appeared to have a low contribution because of its slow rotational speed.

As shown in Fig. 6b, the engine harmonic orders and engine fan noise were dominant in the range of 100–600 Hz. As the engine speed increased, the contribution of the engine 4th harmonic and engine fan order increased. Differential gear sets of the front and rear axles have relatively high rotational speeds but have a small number of teeth, so their generated noise appears as low-order components.

As shown in Fig. 6c, gear whine noise was dominant in the high-frequency range above 1000 Hz. Low-order noise occurs in a gear pair with a low rotational speed. Because the A-weighting factor has a small value below 1000 Hz, a high weighting sound pressure level occurred in a relatively high-speed gear. In particular, the gear of the transfer case, which rotates at the fastest speed in the transmission and transmits power to the front axle, generates the greatest noise.

**Figure 7:**
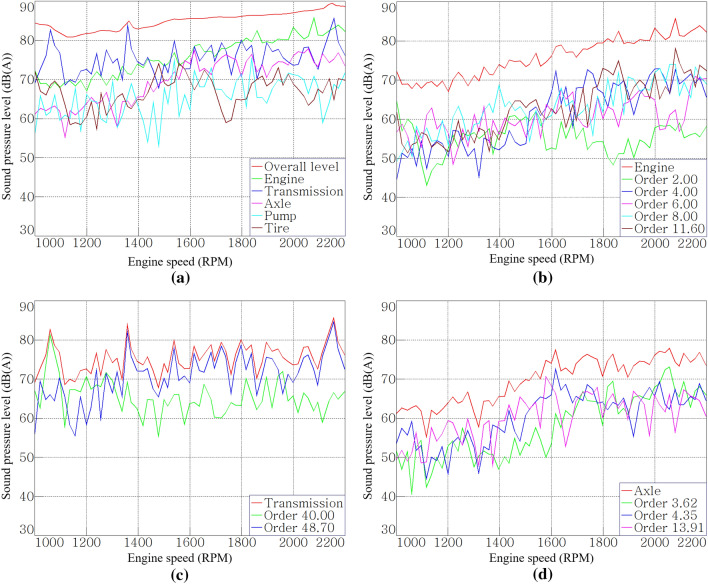
Order analysis results: (**a**) contribution of dominant noise source, (**b**) order contribution of the engine, (**c**) order contribution of the transmission, and (**d**) order contribution of the axle.

Figure 7a shows the overall level of noise inside the cab and the contribution of the sources to the noise inside the cab. Figure 7b–d shows the contribution of each sound source and related order according to Table 3. The contributions of each sound source were calculated by summing the sound pressure of relevant order. In Fig. 7a, the gear whine noise of the transmission was most dominant below 1600 rpm and the engine noise was dominant above 1600 rpm. In other words, the gear whine noise of the transmission and engine noise were the dominant noise sources. In particular for the engine, the contribution increased as the speed increased. Because it had a similar level compared with the overall level in the range above 1600 rpm, the engine was evaluated as the most dominant source.

The diesel engine of the target tractor is a four-stroke engine, and it is known that noise and vibration are generated in the second order of the explosion frequency and its harmonic order^23^. As shown in Fig. 6a,b, the engine’s 2nd, 4th, 6th, and 8th harmonic order and engine fan noise of 11.6th order were judged to be the main noise from the engine. The 4th, 6th, and 8th harmonic orders and the engine fan noise of the 11.6th order had higher contributions than the 2nd order in Fig. 7b. In particular, the 4th order showed a sound pressure level of more than 65 dB(A), and it was the most dominant noise component in the low-frequency range below 200 Hz.

Figure 7c shows the order contributions of each gear mesh of the transmission at stage 16. Gears that do not transmit power due to clutch engagement or disengagement according to the gear stages were excluded from the analysis, because these do not generate high noise under almost no-load conditions. As shown in Fig. 7c, the transfer case gear rotating at the highest speed in stage 16 showed the greatest contribution to the transmission noise. The fact that the high-speed gear, that is, the high-order gear mesh, generates high noise level can also be seen from Fig. 7a, which shows the order contribution of the input gear of hydraulic pump (43rd order only), and Fig. 7d, which shows the order contribution of the axles. It can be seen that a relatively low-order noise occurs on axles with low speeds due to the large reduction ratio of the main shift part and the sub-shift part, and the noise levels were low. On the other hand, in the case of the input gear of the hydraulic pump which receives power from the power take-off (PTO) driveline directly connected to the engine, there is no speed reduction at all, and the maximum noise level of 69 dB(A) was generated in the 43rd order by the speed increase gear with many teeth.

The tractor transmission has a large reduction ratio; therefore, high noise contributions are generated from the gear close to the engine or from the high-speed gear, such as stage 16 in this study. In addition, it can be expected that the input gear of the hydraulic pump, which is directly connected to the crank shaft, and the gears of the PTO driveline generate high-order noise components with high noise levels. Therefore, attention should be paid to gear design with a high GMF for noise reduction.

In addition to the order components that occur in the multiples of engine speed, it was confirmed through Fig. 6c that high noise occurred in the broadband of less than 2000 Hz at 1000 rpm and 1400–2200 rpm. In particular, in the section overlapping the 4th and 48.7th orders with high contribution, a noise of 65 dB(A) or more was generated, as shown in red in the figure. For noise generated in a wide frequency range as described above, it was judged that the application of sound-absorbing and sound-insulating materials that exhibit a noise-reduction effect in a region above a certain frequency would be effective.

**Figure 8:**
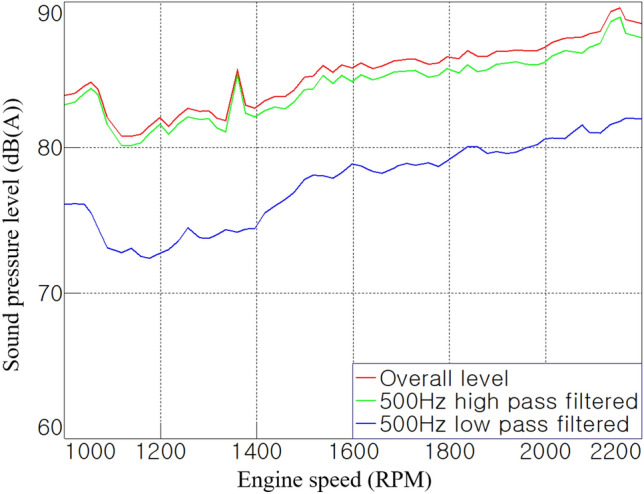
Noise contribution according to the frequency range.

In general, it is known that sound absorption materials have effective performance in the frequency range above 500 Hz^10^. Figure 8 shows the noise contributions of the broadband to which the low- and high-pass filters were applied at 500 Hz. The noise in the range above 500 Hz was found to be close to the overall level, and it had a high level of more than 10 dB(A) compared to the noise in the range below 500 Hz. Therefore, it was expected that acoustic treatment using acoustic materials would be effective for the target tractor cab.

In addition, it is known that the contributions of noise transferred through air-borne paths such as holes and through-paths are significant in the high-frequency range^13^. If this direct path has a size of 1/4 or more of the wavelength according to the frequency of the transferred noise, the sound-insulation performance of the cab deteriorates^16^. Therefore, it can be seen from Fig. 8 that it is important to block the direct paths between the cab and external space.

It was identified that the high-frequency noise significantly contributed to the cab interior noise through order analysis and broadband contribution results in this section. The sound absorption material can absorb acoustic energy in the high-frequency range more effectively than in the low-frequency range, and the acoustic barrier can block the transmission of noise in a band over a certain frequency. Therefore, to reduce the cab interior noise, acoustic energy should be dissipated using sound-absorbing material in a closed space such as a cab or engine room, and the direct paths, such as holes and through-paths, should be well blocked.

### Insulation of direct path

In this study, noise in the frequency range above 500 Hz, which mainly contributed to the cab interior noise, was selected as the reduction target. The high-frequency noise is mainly transmitted through the air-borne path; therefore, it is very important to block direct paths such as holes and through-paths between the cab and the outside.

Table 5 shows direct paths of the cab identified visually. The identified paths were an opening area of 20,150 mm^2^ (130 mm × 155 mm) between the engine room and the dashboard, a hole with a diameter of 50 mm at the rear of the cab, and a hole with a diameter of 7 mm for the parking brake. The opening area between the engine room and the dashboard was blocked by a steel plate of 2 mm thickness. Steel is a nonporous and high-density material that is suitable as an acoustic barrier that should not allow vibrations upon the arrival of sound waves. Additionally, polyurethane (PU) foam sound-absorbing material was applied to the area of 294,822 mm^2^ between the engine room and the cab as shown in the second row of Table 5. The flame-retardant PU foam material is suitable for application to the engine room with high temperature. Moreover, the effective thickness of 50 mm, which is 1/10 of the wavelength of the 680 Hz band, was secured to enable sound absorption in 680 Hz band or higher. This is an additional treatment to minimize unwanted sound build-up or reverberation within the engine room, which is a closed space before sound waves reach the outer surface of the cab. The holes in the rear of the cab and the parking brake were blocked with a dedicated rubber grommet and a rubber case, respectively.

**Table 5:** Acoustic treatment for the direct path of the cabin.

Part	Before treatment	After treatment
Dashboard		
Back side of the seat		
Parking brake		

**Figure 9:**
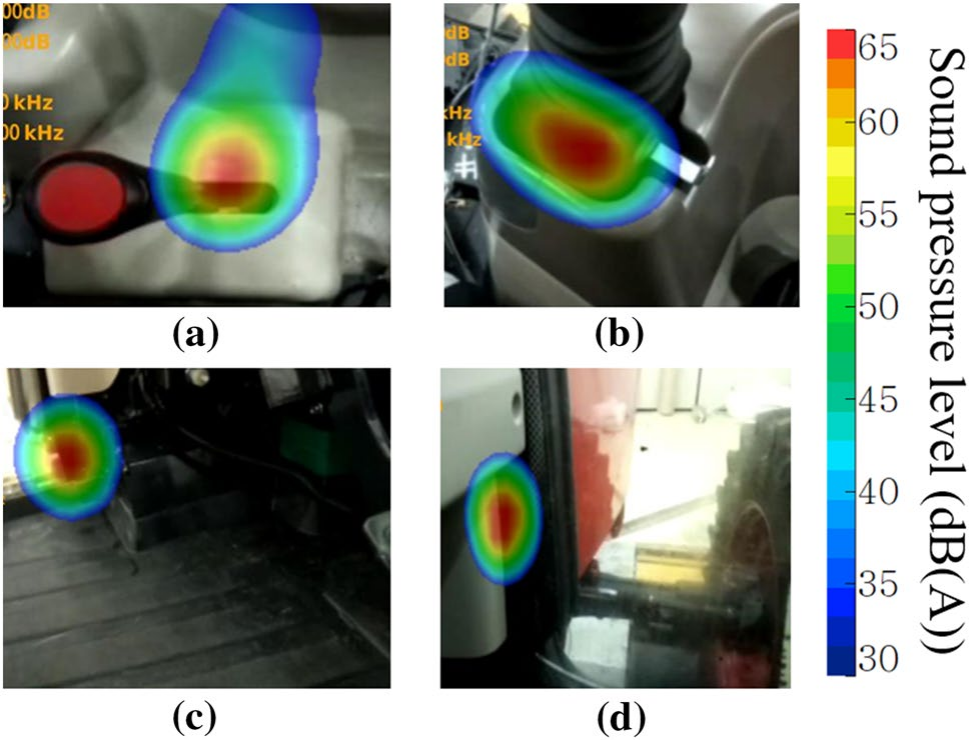
Detected local maximum noise level using sound camera.

In addition to the paths in Table 5, additional paths were identified through acoustic images inside the cab taken with a sound camera. Figure 9 shows the local maximum of the sound pressure level in the cab using the sound camera. It was confirmed that noise was introduced into the gap of the mechanical shift linkage under the creep gear lever and the loader control lever, as shown in Fig. 9a,b. Figure 9c,d show the lower part of the dashboard, which was judged as the noise transmitted from the engine room. In this study, the gap between the mechanical shift linkage and the levers was blocked using an ethylene-propylene diene monomer (EPDM) material, which is often used as a packing and sealing material because of its excellent adhesion during compression. After the treatments, the local maximum on the sound camera did not appear. In actual mass production, it would be desirable to insulate the cab by applying dedicated grommets or replacing the mechanical shift linkages with an electronic shift system.

**Figure 10:**
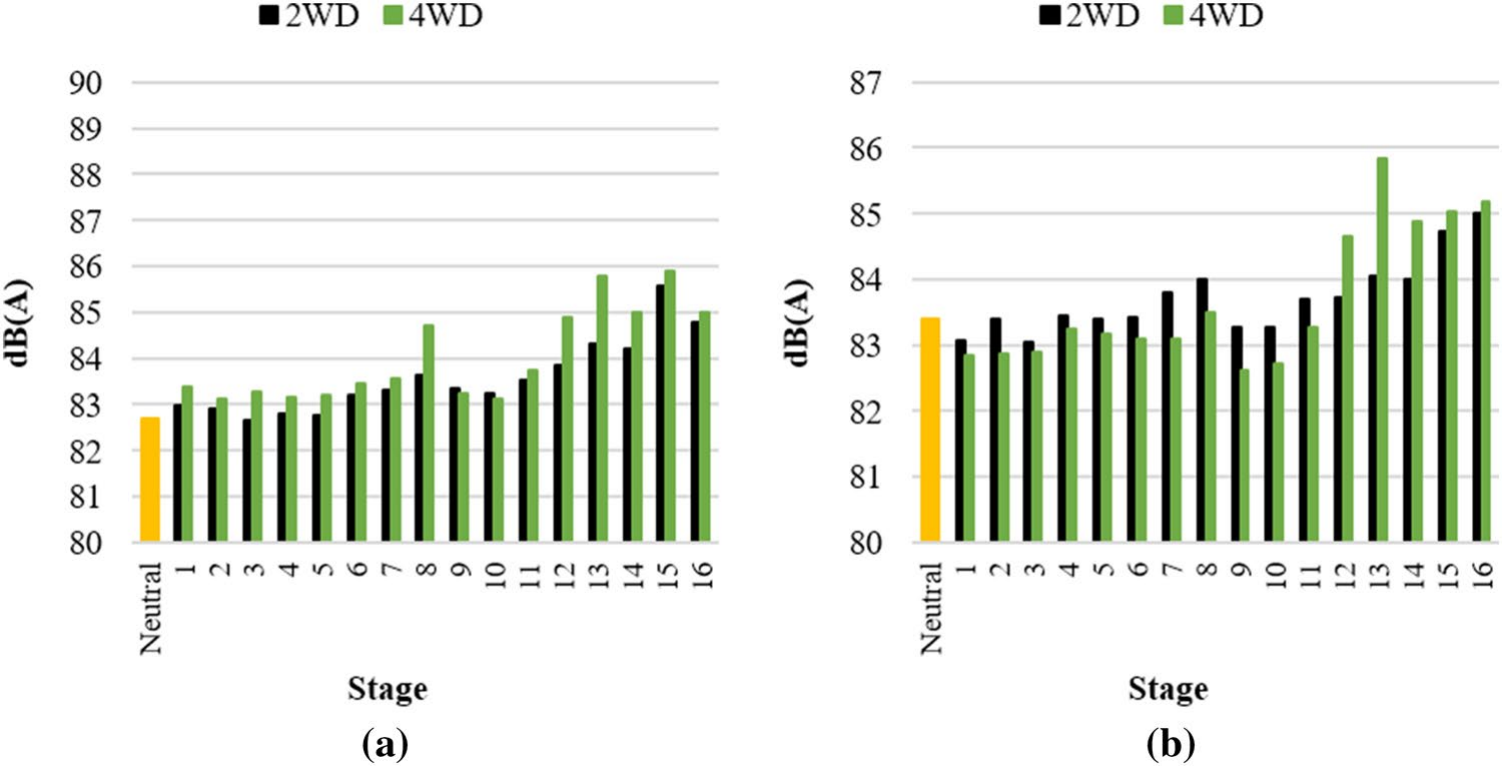
Maximum overall noise level after the treatment: (**a**) operator’s left-ear position, and (**b**) operator’s right-ear position.

Figures 10 shows the maximum noise level according to the gear stage after blocking the direct paths and applying PU foam sound-absorbing material between the engine room and the dashboard. Overall, the noise level of 4–6 dB(A) was reduced after the acoustic treatments. It is noteworthy that a relatively simple treatment that improves the insulation of the cab can reduce the interior noise by 4–6 dB(A). In particular for mechanical transmission, dedicated grommets should be applied to the part connected to the outside of the cab so that there are no gaps, and gear shift boots should be installed at the bottom of the gear lever to minimize the contribution to the interior noise.

**Figure 11:**
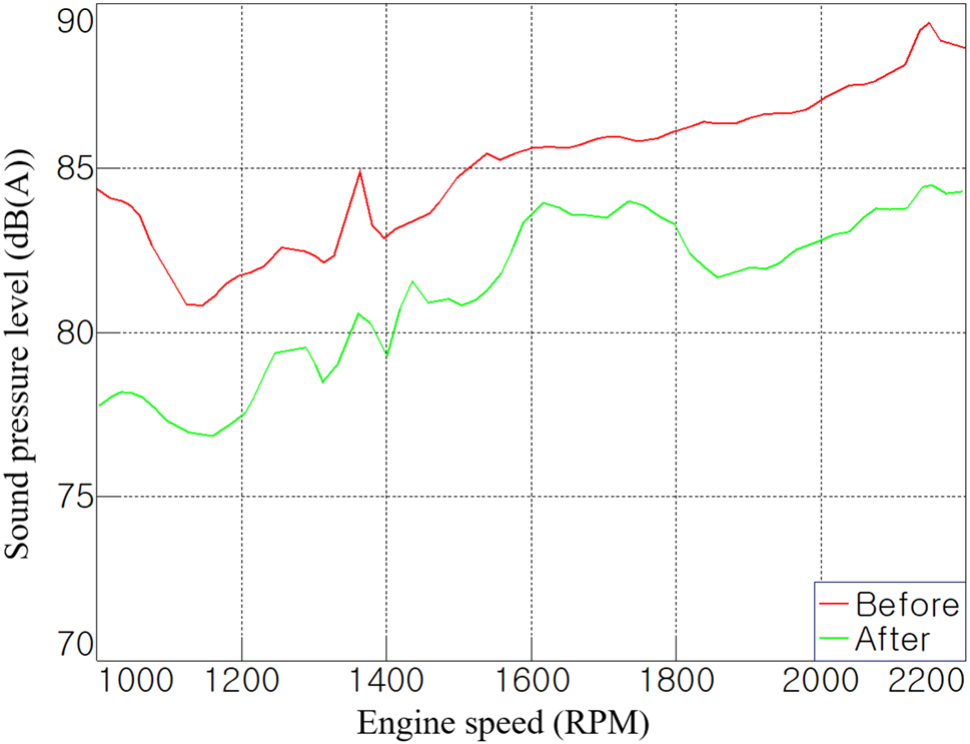
Overall noise level at 4WD stage 16 (RH).

In stage 16, where the highest noise occurred, the noise level was reduced by 2–6 dB(A) over the entire operating range, as shown in Fig. 11. In the 1000–2000 rpm range, a reduction of approximately 6 dB(A) was noticed compared with before the treatment, whereas, at 1600 rpm, a sharp increase in noise level of 3 dB(A) or more was observed during acceleration. It is estimated that noise characteristics that were previously masked by air-borne noise appeared; therefore, additional analysis is required. This booming phenomenon often occurs in the low-frequency range and occurs in various forms so that both structure-borne and air-borne paths can be transfer paths^12,24^. As such, the results in Fig. 11 suggest that even if the overall level is reduced through sound absorption and sound insulation, new noise characteristics that have been previously masked may appear.

**Figure 12:**
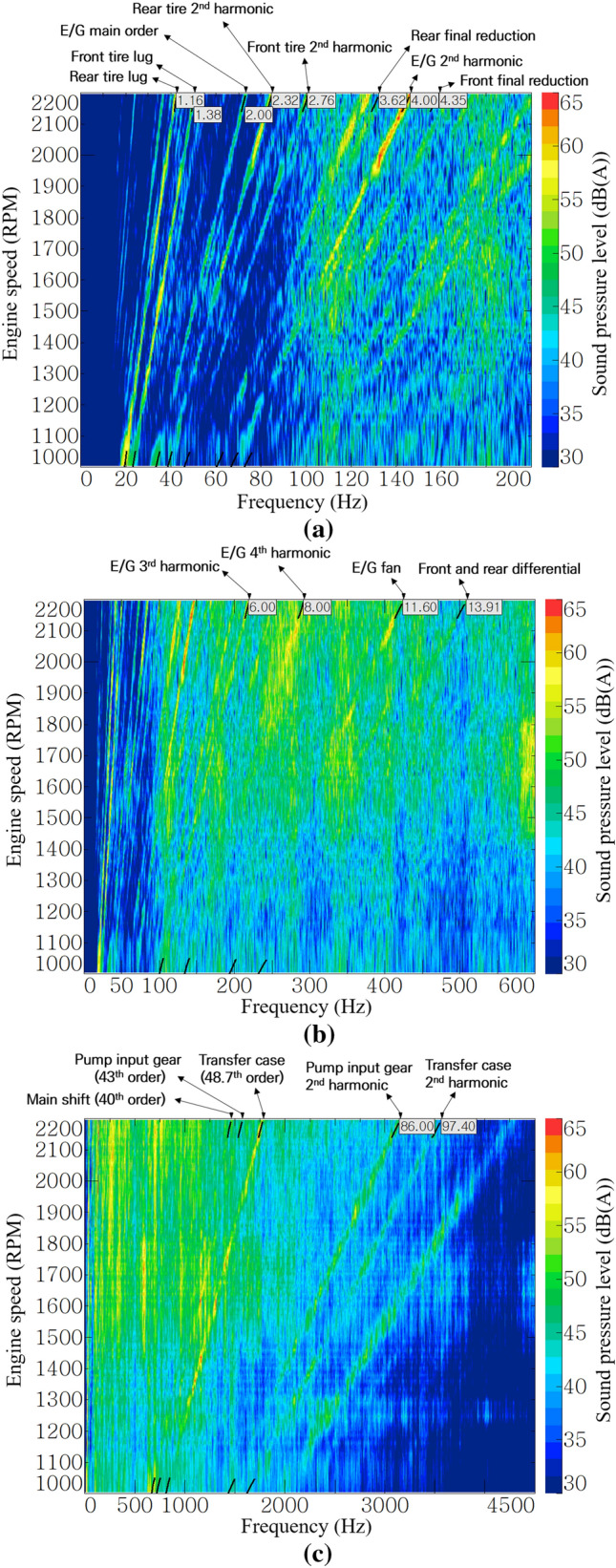
Post-acoustic-treatment noise color map of stage 16 at operator’s right-ear position: (**a**) up to 200 Hz, (**b**) up to 600 Hz, and (**c**) up to 4500 Hz.

This study focused on air-borne noise reduction through sealing the paths between the cab and the exterior, as well as sound insulation between the engine room and the dashboard. Therefore, it is difficult to have an effect on the noise in the low-frequency range. As shown in the frequency range below 200 Hz in Fig. 12a, there was no significant difference compared with Fig. 6a before the acoustic treatment was applied. On the tractor used in this study, low-frequency noise was mainly dominated by excitation due to the engine harmonic order and tire lug pattern. To reduce this noise, a vibration isolator, such as a resilient mount or cab suspension, is required to attenuate vibration in the transfer path or to reduce the excitation force of the sources.

However, the noise-reduction effect was clearly confirmed for engine fan noise, which was mainly transferred through the air-borne path. In Fig. 12b, the sound pressure level of the 11.6th order representing the engine fan noise was reduced near 1850 rpm and over 2000 rpm when compared with Fig. 6b before the acoustic treatment was applied.

**Figure 13:**
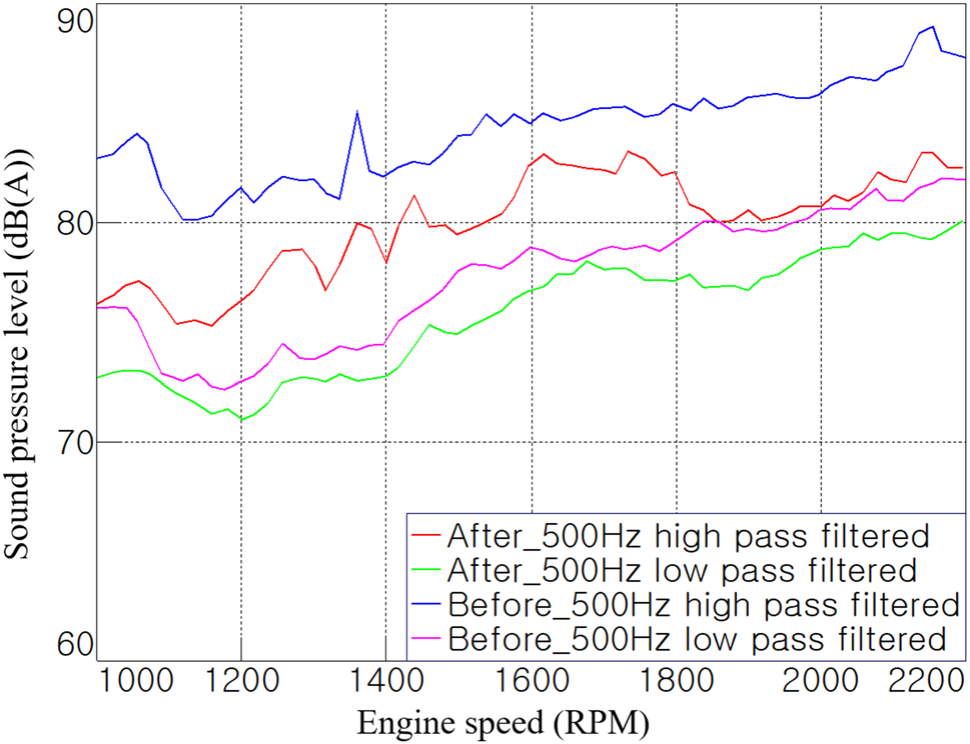
Comparison of broadband contributions.

The main noise reduction was observed in the high-frequency range above 500 Hz, where the sound-insulating material exhibited its performance. In the 1500–2000 rpm section of Fig. 12c, the noise-reduction effect was clearly shown in the region above 500 Hz, compared with Fig. 6c before the acoustic treatment was applied. Figure 13, which applies a band pass filter based on 500 Hz, clearly shows the effect of the acoustic treatment in this study. In the range below 500 Hz, a noise reduction of approximately 1–2 dB(A) was noticed overall, whereas, in the range above 500 Hz, a noise-reduction effect of at least 2–6 dB(A) is shown. In other words, the overall noise level was also mostly reduced in the high-frequency range.

**Figure 14:**
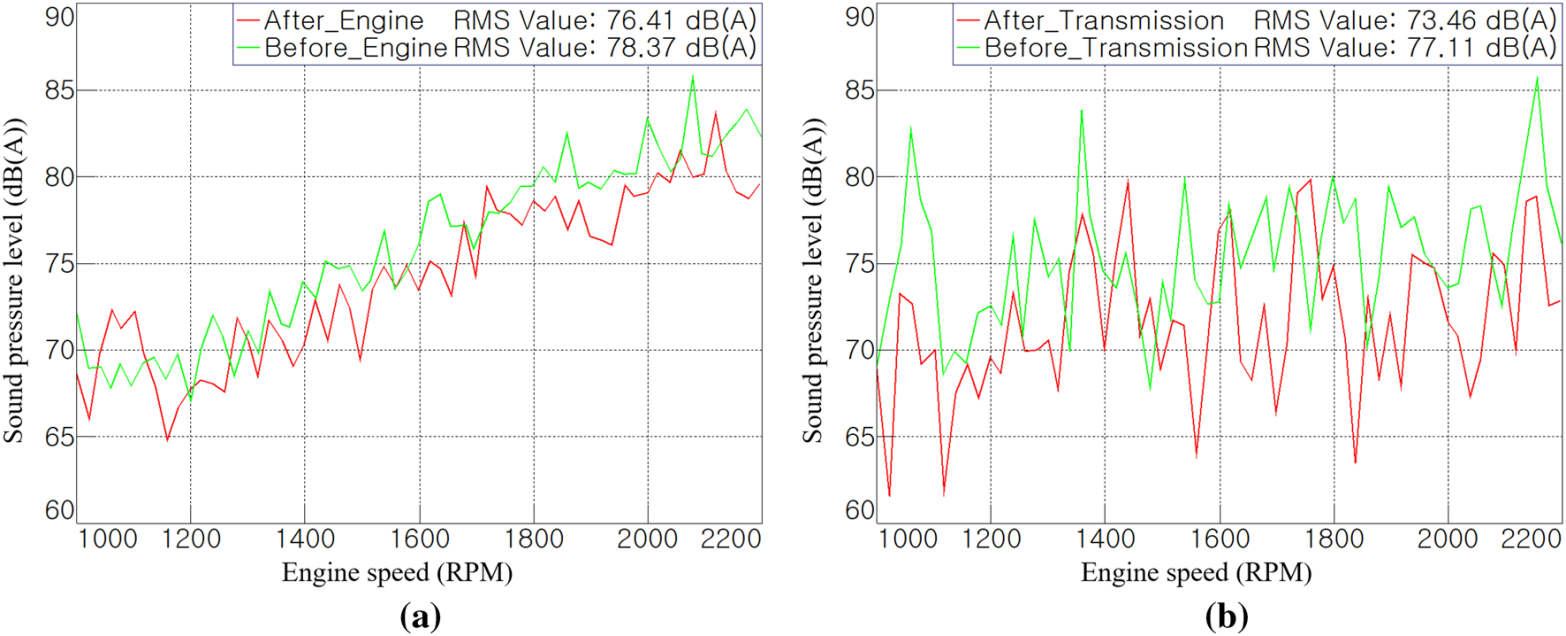
Order analysis results after the treatment: (**a**) comparison of engine order sum, and (**b**) comparison of transmission order sum.

Figures 14 shows the noise-reduction effects on the engine and transmission, analyzed as having the largest contribution to the cab interior noise. For the engine, the tendency of the sound pressure level to increase according to the rotational speed was maintained, and it was reduced by approximately 2 dB(A) based on the root mean square (RMS) value during run-up. In the entire operational area, the transmission noise was reduced by approximately 3.6 dB(A) and appeared to be less than 80 dB(A). Compared to the engine harmonic orders (2nd, 4th, 6th, and 8th) and fan order (11.6th), the gear mesh orders (40th, 48.7th) of transmission occurred in the high-frequency range; hence, the acoustic treatment in this study was more effective to the transmission noise. This also means that the contribution of noise radiating from the tractor transmission and entering the cab through the air-borne path is significant.

## Conclusions

The cab interior noise, which is an important performance indicator of tractors, has been studied, mainly focusing on the reduction of structure-borne noise in the low-frequency band. This is to reduce the boom noise, which represents a severe noise increase in a specific operating condition. Although many studies have consistently highlighted that the most dominant noise in the entire operation of the tractor is air-borne noise, and the insulation of the cab is important for its reduction, there have been few studies on air-borne noise reduction in the high-frequency band.

Therefore, this study focused on identifying and reducing air-borne noise, an important component of the cab interior noise, through a chassis dynamometer test. In particular, the noise-reduction effect was investigated by blocking the direct paths of the cab, such as gaps and holes, and insulating the area through which the engine transmission noise was transferred.

In summary, the noise contribution of each noise source to the driver’s ear position was analyzed in this study, using an order analysis to reveal the current state of the tractor used. Based on the results of the order analysis, the direct paths between the cab and exterior were blocked to reduce the air-borne noise in the high-frequency range, and the engine transmission noise was reduced using acoustic material. Finally, by improving the sound insulation of the cab, the noise-reduction effect was confirmed.

The total opening area of 22,152 mm^2^ of the target tractor cab was sealed, and the PU foam sound-absorbing material was attached to an area of 294,822 mm^2^ between the engine room and the dashboard. The cab interior noise was reduced in the frequency band above 500 Hz, and a noise reduction of 4–6 dB(A) was confirmed in the entire operating range. This is equivalent to reducing the sound pressure inside the cab by half.

In this study, it was confirmed that the contribution of the direct path and noise transferred from external noise sources was high. Therefore, to reduce the cab interior noise in the entire operating range, the sound insulation of the cab should be improved first. The cab interior noise reduces the operator’s work efficiency and is a performance indicator of agricultural machinery, it is likely to affect consumers’ purchasing decisions. From the results of this study, it is expected that the improvement of the cab insulation will reduce the tractor cab interior noise and contribute to the improvement of the farmworker’s work efficiency and convenience.

## Data Availability

The datasets during and/or analyzed during the current study available from the corresponding author on reasonable request.
